# Laparoscopic Castration Using Bipolar Forceps vs. Orchiectomy in Dogs: A Comparison of Two Techniques

**DOI:** 10.3390/ani11113041

**Published:** 2021-10-24

**Authors:** Inês Tenreiro Tavares, Ramón R. Barreno, José P. Sales-Luís, Carlo G. Vaudano, José Raduan Jaber

**Affiliations:** 1 Faculty of Veterinary Medicine, Lusófona University, 1749-024 Lisboa, Portugal; 2 Faculty of Veterinary Medicine, Autonomous University of Ciudad Juarez, Cd Juárez 32315, Mexico; ramriver7@hotmail.com; 3 Centre for Interdisciplinary Research in Animal Health, Faculty of Veterinary Medicine, Lisbon University, 1300-477 Lisboa, Portugal; jpluis@fmv.ulisboa.pt; 4 Surgical Department, Oeiras Veterinary Hospital, 2780-176 Oeiras, Portugal; c.vaudano@hvo.com.pt; 5 Departamento de Morfologia, Facultad de Veterinaria, Universidad de Las Palmas de Gran Canaria, 35413 Las Palmas de Gran Canaria, Spain; joseraduan.jaber@ulpgc.es

**Keywords:** dog, laparoscopic castration, bipolar electrocoagulation, pain, PCR, UMPS, salivary cortisol, serum cortisol

## Abstract

**Simple Summary:**

Castration of dogs is one of the most often performed surgeries in veterinary medicine. Minimally invasive techniques used in human medicine are now being used in animals. We compared the feasibility and effects on pain and inflammation of a new laparoscopic technique with the classical castration technique. The animals in which the new technique was applied showed less pain and inflammation then the other group. Our results suggest that this is a feasible alternative to classical castration.

**Abstract:**

This paper aimed to study the feasibility of a new laparoscopic castration technique in male dogs, evaluate the pain associated with it, and compare it with the classical orchiectomy. Surgical times, pain scores, blood and salivary cortisol, and CRP were recorded and compared between the two groups. The use of high-frequency bipolar forceps allowed quick and uneventful laparoscopic procedures. The laparoscopic group had significantly lower pain scores, cortisol, and PCR values than the orchiectomy group. No complications were seen in any group. Our results suggest that this laparoscopic castration is a safe and beneficial surgical alternative to traditional orchiectomy in dogs.

## 1. Introduction

Elective sterilization of dogs is one of the most performed procedures in veterinary practice, which is reported to increase dog’s life expectancy [1,2]. It is used for contraception, to control dog overpopulation, to prevent diseases [2], and to reduce unwanted sexual and aggressive behaviors [3]. To date, an array of spaying procedures has been described, including castration by open or closed orchiectomy [4], scrotal ablation [5], vasectomy, vasal occlusion with calcium chloride [6], laparoscopic vasectomy and sterilization [7], and bilateral vasocystostomy [8].

In male dogs, the most often used technique is an orchiectomy, in which the testis is moved cranially and exposed with a pre-scrotal incision in order to be removed [9]. This conventional method for castration is associated with several postoperative complications such as scrotal swelling, wound dehiscence, infection, maggot infestation, and hemorrhage [10]. Hemorrhage can lead to scrotal hematoma or intra-abdominal hemorrhage. These may necessitate surgical repair by scrotal ablation and locating and ligating the spermatic cord [9]. Additionally, postoperative care of the animal is required to avoid infection [11].

In recent years, minimally invasive surgery has developed rapidly both in human and veterinary medicine, and many new techniques and instruments have been created. [12] Laparoscopic surgeries are becoming more common in veterinary medicine due to their benefits, such as reduced postoperative pain, reduced incidence of surgical site infection, shorter hospital stays [13,14,15,16], much smaller wounds that require no postoperative care dressings [10], and lower morbidity [17]. As pet owners become more aware of these techniques, they await a minimally invasive surgery to be an option for their pets [4].

So far, laparoscopic castration is considered to be a suitable choice if combined with another surgery with a laparoscopic approach, such as cystopexy [18], deferentopexy [19], colopexy [20], and gastropexy [21]. Besides this, it can be applied to criptorchidectomy and neoplastic testes in dogs [22] and as an option for mass sterilization programs in overpopulated urban and industrial areas [10].

To date, there are few references regarding laparoscopic sterilization (LS) of male dogs with descended testis. The first study on this used the occlusion of the ductus deferens, resulting in rapid azoospermia but maintained libido and mating behavior. The development of this technique had the goal of sterilizing the male animal without changing its physical characteristics or endogenous hormone secretion [23]. Other studies described a technique in which a segment of the vas deferens was cauterized and resected with monopolar grasping forceps [11] or a laparoscopic-assisted colopexy and sterilization, where both vessels and the deferens were double clipped and then coagulated, and the tissues in the area between the clips cut [20]. The most recent report of a laparoscopic sterilization technique was performed by Mahalingam et al., where besides cauterizing and resection of 2 to 3 cm of vas deferens, the spermatic artery–vein plexus was double clipped [7].

Thus, this study aimed to evaluate the feasibility of a new technique of LS in healthy dogs. We also intended to assess differences in time of surgery and pain responses between orchiectomy (OR) and the LS method presented here. We hypothesized that LS would be less painful than OR because of smaller incisions and decreased soft-tissue trauma. To test our hypothesis, we evaluated pain using biochemical responses and subjective measurements in two groups of dogs sterilized by either OR or LS.

## 2. Materials and Methods

### 2.1. Animals

A total of twenty healthy stray male dogs from a shelter of different breeds, mixed-breeds, and ages were enrolled. (see Table 1).

Inclusion criteria were that the dogs had good body condition, suitable temperament, and were not receiving concurrent medications. Dogs were considered healthy based on history, complete physical examination, hematological examination, and serum biochemical analysis on admission, and therefore classified as American Society of Anesthesiologists (ASA) category ASA 1.

The Ethical Committee approved the study protocol of the Faculty of Veterinary Medicine of Lisbon University. Dogs were randomly assigned to two groups of ten animals, pre-scrotal orchiectomy (OR) or laparoscopic sterilization (LS). Food was withheld for 12 h and water for 8 h before surgery. The animals were admitted the day of the surgery. A consent document advising of the risks of each procedure, especially for laparoscopic sterilization and the possible need to convert to an open celiotomy for situations such as uncontrollable hemorrhage or iatrogenic injury, was signed by the dog’s caretakers. The procedures were performed under the Portuguese Government for Animal Care Guidelines (DL No 260/2012).

### 2.2. Surgical Procedures

Before surgery, a 25 mm, 22-gauge catheter (Introcan-W; B. Braun, Lisbon; Portugal) was placed in the cephalic vein for drug administration and collection of blood samples. Peripheral venous blood and salivary samples were collected one hour before induction of anesthesia to obtain pre-operative samples (baseline values).

The anesthesia protocol was the same for both groups. The dogs were premedicated with 0.05 mg/kg acepromazine (Calmivet; Vetoquinol; Lisbon, Portugal) and 5 mg/kg intravenous tramadol (Tramadol; Labesfal; Lisbon, Portugal). Anesthesia was induced with 4 mg/kg intravenous propofol (Propofol Lipuro; B. Braun; Lisbon, Portugal) and maintained with isoflurane (Vetflurane; Virbac; Portugal) in 100% oxygen (100% Medicinal Oxigen; Conoxia; Lisbon, Portugal) delivered through a non-rebreathing circuit. The dogs were closely monitored during the surgical procedure with pulse oximetry, electrocardiography, non-invasive blood pressure, capnography, and temperature.

### 2.3. Orchiectomy

The conventional open castration by the standard pre-scrotal method [5] was made using a 2-0 absorbable synthetic monofilament glyconate suture (Monosyn; Braun; Lisbon, Portugal). Recovery from anesthesia was uneventful.

### 2.4. Laparoscopic Castration

The laparoscopic castration used bipolar forceps with integrated scissors (5 mm, RobiPlus; Karl Storz; 78532 Tuttlingen, Germany). The technique applied was novel since we did not apply clips [7].

**Figure 1:**
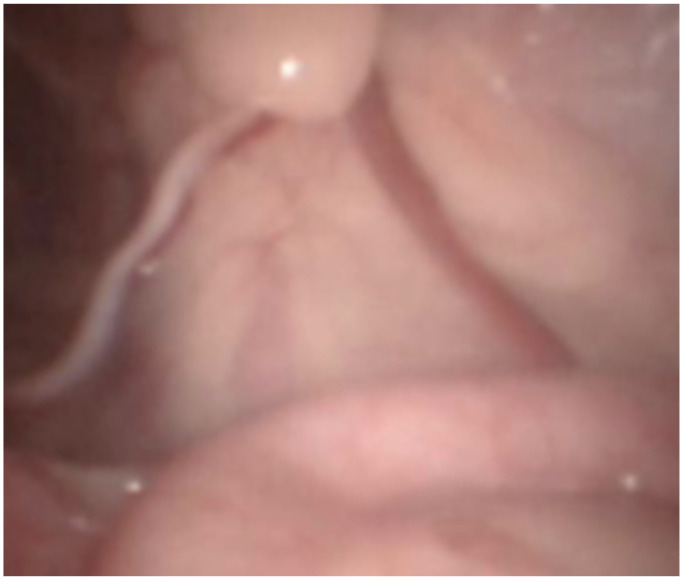
Laparoscopic anatomy of the vas deferens (black arrow), and the artery and vein plexus (white arrow) at the entrance of inguinal canal.

**Figure 2:**
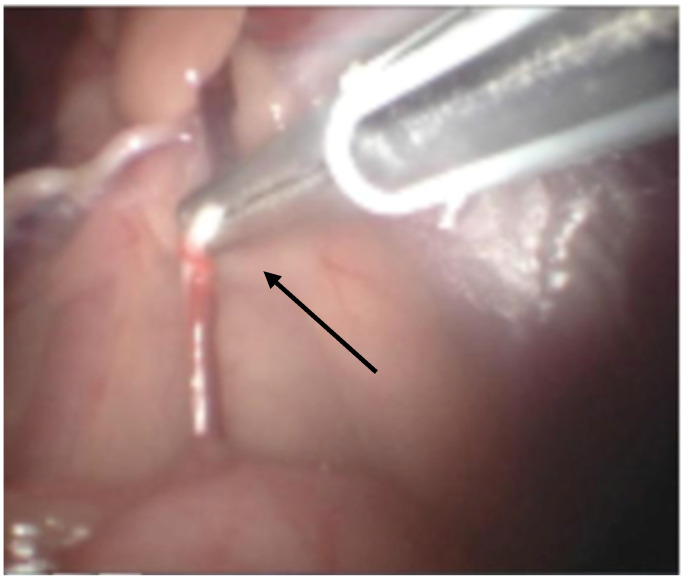
Coagulation of artery and vein with the high-frequency bipolar forceps with integrated scissors (arrow).

**Figure 3:**
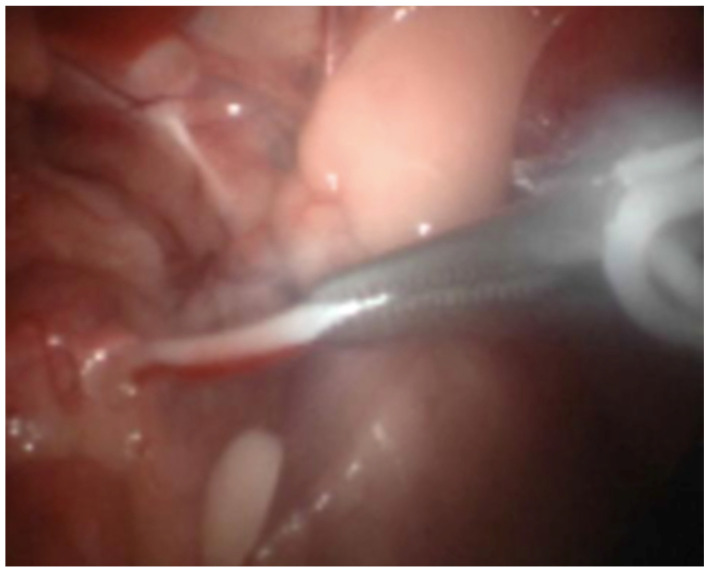
Image of the laparoscopic surgery showing the coagulation of vas deferens.

**Figure 4:**
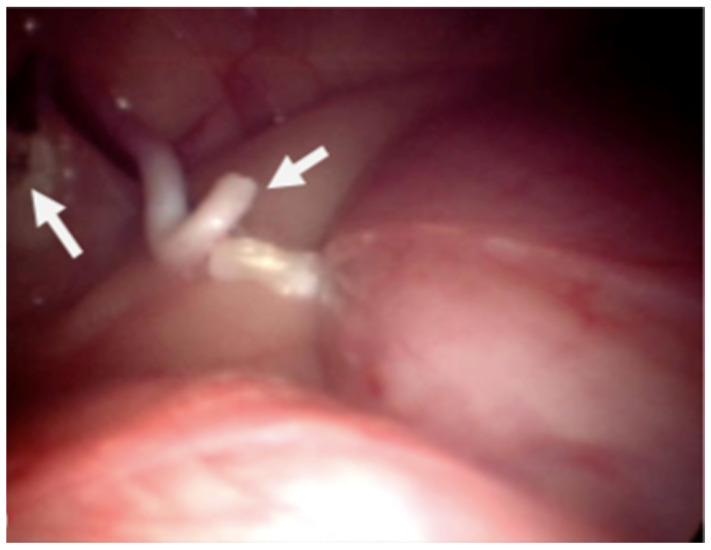
Image of the laparoscopic surgery to confirm that both structures were cut, and without the presence of active hemorrhage.

**Figure 5:**
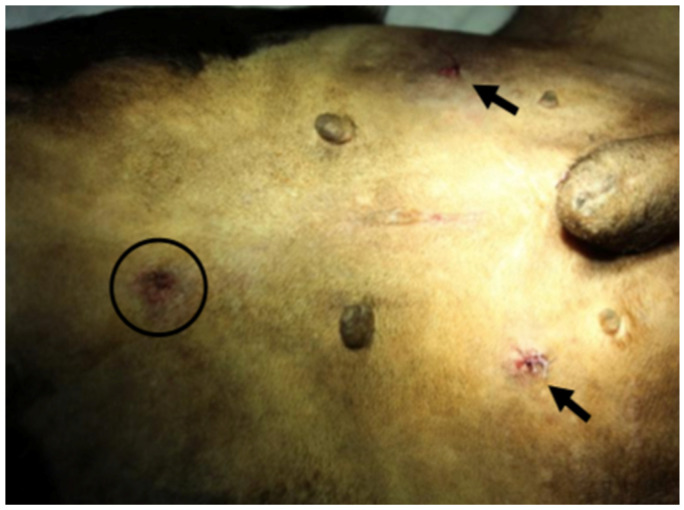
View of the sutures in both trocar sites (arrows) and Veress needle incision (circle).

Dogs were placed in dorsal recumbency on the surgical table without being tied down. The bladder was emptied by catheterization. The pneumoperitoneum was established with a Veress needle (2.1 mm; Richard Wolf, 75438 Knittlingen; USA) inserted caudally to the xiphoid process for abdominal access. CO_2_ administration was provided via automatic insufflator (Electronic Insuflator 2002; Cabot Medical; USA) with a gas flow of 9 L/min to a pressure set at 9 to 11 mmHg. Once the pneumoperitoneum was established, a stab incision was made through the skin and exposed the peritoneum. A perimeter mark was made with the cannula to achieve the incision length. The first cannula (threaded, 5.5 mm in all cases) was placed in the incision. Then, we inserted the telescope (5.3 mm; 0°; Panoview; Richard Wolf; 75438 Knittlingen, USA) using a standard clockwise rotation to evaluate the abdomen and iatrogenic injuries. Later, we positioned another cannula on the opposite side of the telescope port and placed the animals in Trendelenburg position. High-frequency bipolar forceps with integrated scissors (5 mm, RobiPlus; Karl Storz; 78532 Tuttlingen, Germany) were introduced through the left instrument port to handle the vessels and the deferens of the left testis without previous dissection (Figure 1). The structures were coagulated multiple times along 2–3 cm and cut (Figure 2 and Figure 3). We applied the same procedure to the right testicular vessels and deferens. Occlusion of the ductus deferens was considered complete when the structure was coiled and blanched (Figure 4). The portal valves were opened to evacuate the CO_2_ partially, and with a lower pneumoperitoneum pressure, we inspected the abdominal to evaluate the presence of bleeding. After this verification, all the gas was evacuated, and the dogs were placed in normal recumbency. The cannulas were removed just before the suture application to guarantee maximum gas evacuation. Abdominal incisions were closed using a 2/0 absorbable synthetic monofilament glyconate suture (Monosyn; Braun; Lisbon, Portugal) in a simple interrupted suture pattern, and the skin was closed with an intradermal pattern with a single absorbable 3-0 suture (Monosyn; Braun; Portugal) (Figure 5). No dressings were applied.

After the surgical procedures, dogs received a single dose of meloxicam 0.2 mg/kg subcutaneously (Metacam; Boerhinger Ingelheim; Lisbon, Portugal) and were carefully monitored. The dog had a daily clinical examination, which included pain assessment and samples collection. Further meloxicam was administered the following two days (0.1 mg/kg subcutaneously). All the dogs were discharged home two days after surgery. One week after surgery, the animals returned to the veterinary hospital for a physical examination, evaluation of proper wound healing, and collection of blood samples.

### 2.5. Recorded Variables and Postoperative Pain Assessment

Data recorded included breed, age, body weight, duration of surgery, and occurrence of intraoperative bleeding or surgical complications.

The severity of pain was monitored 1, 12, and 24 h after surgery based on pain scores obtained with the UMPS (University of Melbourne Pain Scale). This is considered a validated method for assessment of postoperative pain in dogs [3]. It consists of multiple descriptors arranged in six categories which include behavioral and physiologic responses, and some observer bias can be eliminated by weighting of certain behaviors [4]. The score could vary between zero and twenty-seven (0–27). Pain was assessed by only one person familiar with the pain scoring system and semi-blind to the procedure. A pain score > 10 was considered justification for rescue analgesia, and methadone (0.5 mg/kg) would be administered intravenously if necessary. Nonetheless, we did not administer methadone in any of our animals since they did not reach a pain score of 10 in the UMPS.

Besides this, blood and salivary samples were collected to evaluate postoperative pain. Hence, we analyzed blood and salivary cortisol at 1, 12, 24 h; C-reactive protein (CRP) was evaluated in blood samples collected 24 and 168 h (7 days) after surgery to investigate the systemic inflammatory response to the surgical procedures. We also looked for a correlation between the blood and salivary cortisol values.

Since the surgical procedures were performed at night, dogs slept all night uneventfully. Therefore, a limited number of time points for blood and saliva samples and pain score acquisition were used. The objective was to limit disturbing the dogs and consequently limit potential confounding of the stress/pain variables, as others have previously limited [24].

### 2.6. Statistical Analysis

Data analysis was performed using SPSS statistical software (Version 22, SAS, Buckinghamshire, SL7 2EB, UK). As our data involved the analysis of repeated measurements, we used one-way repeated measures ANOVA to analyze all the variables evaluated and compared between groups for each data collection time point. Significance was set as *p* < 0.05.

## 3. Results

### 3.1. Surgical Procedures and Clinical Follow-Up

A total of 20 patients were randomized and allocated into one of the two groups (OR, LS). There were no significant differences in mean group ages: OR 3.3 years; LS, 1.04 years. There were no significant differences in mean group weights: OR 14 kg, LS 14.1 kg, or the duration of surgery between the OR and the SL groups (see Table 2)

Surgical complications were not found in the OR and LS groups. Conversion to an open approach was unnecessary in the LS group. All dogs recovered uneventfully from anesthesia and were bright, active, and with full appetite at discharge.

### 3.2. UMPS (University of Melbourne Pain Scale)

UMPS was scored by assigning a maximal score of 27 and a minimum score of 0. All dogs had scores of 0 preoperatively. None of the animals in either group had a pain score requiring additional postoperative analgesia (i.e., >10 of a possible 27) at any time after surgery.

**Figure 6:**
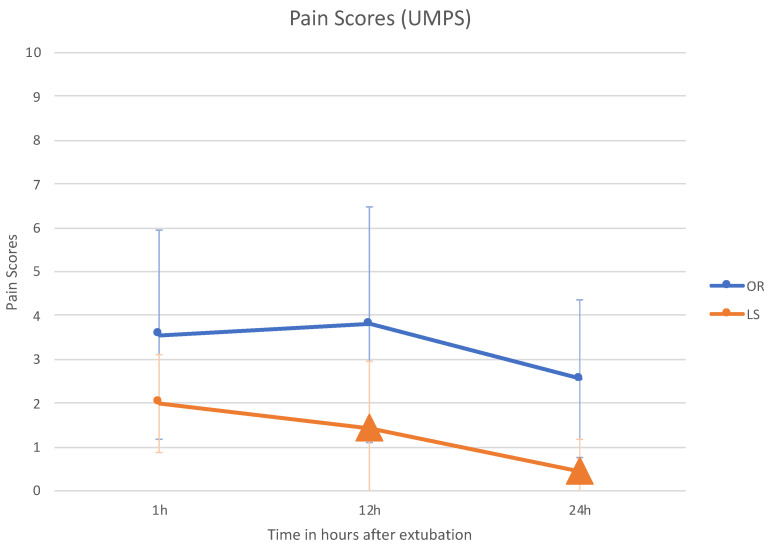
University of Melbourne Pain Scale post-operative scores in groups OR and LS (triangles show the significant values).

The LS group had the lowest pain scores at all postoperative time intervals. UMPS scores were significantly lower for the LS group compared with OR at 12 h (*p* = 0.010) and 24 h (*p* = 0.007) after surgery (see Table 3 and Figure 6).

### 3.3. Serum Cortisol

Preoperative serum cortisol concentrations were within the reference range of 20–250 mmol/L in the two groups and were not significantly different. The mean serum cortisol concentration peaked at 1 h after surgery and returned to baseline 12 h after surgery. Cortisol concentration was significantly lower in the LS group (119 nmol/L; *p* = 0.003) than in the OR group (234 nmol/L) one hour after surgery. No significant differences were found between groups at any other time (see Table 4).

### 3.4. Saliva Cortisol

Preoperative mean saliva cortisol concentrations were not significantly different between groups. The mean saliva cortisol concentration peaked at 1 h after surgery and returned to baseline at 12 h after surgery, following the mean serum cortisol variation trend at the same time intervals. The mean saliva cortisol concentration was significantly lower for the LS group (11.1 ng/mL; *p* = 0.039) than the OR group (15 ng/mL) at one hour after surgery. A strong correlation (r = 0.58) was found between serum and saliva cortisol concentrations. No other significant differences between groups were found at any time (see Table 4).

### 3.5. C-Reactive Protein (PCR)

Preoperative mean PCR concentrations were not significantly different between groups. The mean PCR concentration peaked at 24 h after surgery and returned to baseline at 168 h. PCR concentration was significantly lower in the SL group (5.8 μg/mL; *p* = 0.037) than in the OR group (20.8 μg/mL) one week after surgery (168 h). No other significant differences between groups were found at any time (see Table 5).

## 4. Discussion

To the best of the authors’ knowledge, this laparoscopic sterilization technique for dogs is described for the first time in this study. Furthermore, a controlled study has not previously shown postoperative pain assessment after laparoscopic sterilization in dogs. Several complications are associated with laparoscopic surgery, such as viscera perforation, tissue damage caused by energy application, abdominal cavity access, and pneumoperitoneum [25]. Generally, the less experienced and trained the surgeon is, the more frequent the complications [26]. In this study, the surgeon’s experience and the bipolar instrument used contributed to surgical procedures without complications.

Results of the present study show that the surgical technique was simple, feasible, and quick to perform without intraoperative complications or abnormalities requiring conversion to open laparotomy. The execution of both procedures did not differ significantly by time. The OR surgery requires several sutures, whereas, in LS, the most time-consuming step is the trocar placement and pneumoperitoneum establishment.

The Veress needle was used successfully to maintain the pneumoperitoneum through all the laparoscopic surgeries. It was introduced carefully into the abdominal wall without visceral damage due to a tight manual elevation before its introduction. Despite the risk of visceral damage, we consider using the Veress needle easy and less traumatic, and it avoids the difficulties associated with the modified Hasson technique. [27]

Trendelenburg positioning enabled the displacement of the digestive organs towards the diaphragm area, allowing precise observation of reproductive structures. The trocars placement permitted inspection of the abdominal cavity and a broad view of and access to the pelvic organs and tissues. The use of only two trocars had the following advantages: surgery was performed by a solo surgeon and with minor surgical trauma to the abdominal wall, which contributes to less post-operative pain [24].

The high-frequency bipolar forceps used were effective, user-friendly, and safe in coagulating and cutting vas deferens and artery and vein plexus. Since the scissors are integrated into the instrument, fewer surgical maneuvers and instruments were needed, reducing surgical time. Like other sealing and cutting instruments, the RobiPlus caused minimal smoke, resulting in a slight brief loss of observation in the surgical field [23].

Contrary to previous methods described for hemostasis of the spermatic cord, in this technique, no foreign material (metallic or plastic clips) was used and left in the animal. The sutures applied in the wounds from trocars proved to be entirely sufficient. The animals did not show interest in the wounds after surgery. The dog caretakers reported their well-being and high activity level, confirming the short period of convalescence needed due to low level of pain in the abdominal integument.

The aesthetical appearance was another advantage of laparoscopic sterilization. Although the evolution of testicular volume was not measured in detail in our study, we detected, as Mathon et al. observed, that the testicular volume was increased seven days after surgery and decreased by 90% at the end of 60 days [20]. This testicular atrophy happens due to ischemic degeneration [20], indicating a loss of function and a complete castration. This surgical technique allows the male dog to maintain the testis in the scrotum, although in a smaller size, which may pose a more attractive surgical option to male owners, who often avoid the castration of dogs [28].

Concerning postoperative pain, we found that LS was less painful in dogs when compared with OR surgery. Pain scores and cortisol measurements were considered effective in assessing pain levels. The use of multiple methods to assess pain in animals is accepted as the best way to avoid the unbalanced weighing of any single subjective or objective measurement [29,30]. Hence, no gold standard has been established for pain measurement in veterinary patients [31]. Objective physiologic (heart rate, respiratory rate, temperature) and biochemical (cortisol) measurements are usually used as indirect measures of pain, but they give inconsistent assessments of pain if not used with simultaneous evaluation of behavioral changes [32,33,34,35,36].

The measurement of cortisol values has been helpful as an objective indicator of animal pain [34,37,38,39], and it has been described as significantly increasing due to surgical stress in dogs and cats [33,34,35]. Similar results were observed in our study, where a correlation was found between serum and salivary cortisol concentrations. As salivary cortisol is highly correlated with serum cortisol in dogs, and its sampling is non-invasive [40], it has been chosen to measure stress response in these animals [40,41,42]. According to other laparoscopic sterilization studies in dogs [15,43], the first and highest postoperative peak of cortisol in this study occurred one hour after the surgery. Hence, the mean salivary and serum cortisol concentrations in the LS group were significantly lower than in the OR group. Since the anesthesia protocol was the same for every group, anesthesia alone apparently would not explain the difference in cortisol concentrations at 1 h postoperatively. Thus, it is possible that the low cortisol concentrations at hour one in the LS group may genuinely reflect the lower level of pain from the less traumatic surgical procedure.

In dogs, increases in serum CRP concentrations have been described after different surgical procedures; the more severe the tissue trauma made, the higher the magnitude of CRP increase [44,45]. Data confirm that canine serum CRP can be used to differentiate variable degrees of inflammatory activity after elective surgical procedures [46]. At the time of suture removal—usually seven days after surgery—CRP concentration is considered a more useful tool to monitor postsurgical progress than WBC counts in surgical procedures without clinical complications [44,47]. In this study, the use of meloxicam (non-steroidal anti-inflammatory drugs—NSAIDs) could also influence changes in serum CRP. However, it is known that CRP production is mainly dependent on pro-inflammatory cytokines, and NSAID only regulates prostaglandin synthesis [46].

As seen in another study [48], the maximum peak CRP concentration was 24 h in our groups. The CRP concentrations were lower at all times in the LS group, but only at 168 h after surgery, did we observe a significant difference between the CRP concentrations. Other studies comparing inflammation between open and laparoscopic procedures also found significantly lower concentrations of CRP for laparoscopic procedures [46,49,50]. The present data confirm that laparoscopic castration in dogs induces less inflammation, as it is a surgical procedure that produces less tissue trauma.

## 5. Conclusions

In conclusion, the novel laparoscopic sterilization technique was minimally invasive and allowed excellent visualization of the reproductive structures. Significant differences were found in pain-associated variables in the laparoscopic group at one hour postoperative, suggesting that this group had less pain. The surgeries did not have any complications, and the animals recovered uneventfully. Therefore, the authors believe that this laparoscopic castration is a safe and beneficial surgical alternative to traditional orchiectomy in dogs. In the future, it would be interesting to perform further studies, including more time points for the pain analysis—both with UMPS score and cortisol.

## Figures and Tables

**Table 1:** Number of animals by breed in groups OR and LS.

	Number of Animals by Breed			
	Mixed-Breed	Miniature Poodle	Labrador	Yorkshire Terrier
OR	6	2	1	1
LS	10	-	-	-

**Table 2:** Patient characteristics and intraoperative variables (values in mean, min, max).

Patient Characteristics and Intraoperative Variables	OR	LS
Age (years)	3.3 (0.6–7)	1.04 (0.4–2)
Weight (kg)	14 (4.5–27)	14.1 (5.7–24.4)
Duration of surgery (hours)	41.4 (36–49.8)	42 (30–75)

**Table 3:** UMPS Scores (mean + SD) of dogs that underwent orchiectomy and laparoscopic sterilization.

Variable	Group	Time after Extubation		
		1 h	12 h	24 h
UMPS Score	OR	3.56 ± 2.4	3.8 ± 2.68	2.55 ± 1.8
	LS	2 ± 1.12	1.44 ± 1.5 *	0.44 ± 0.73 *

* significant values.

**Table 4:** Mean ± SD values of serum and salivary cortisol recorded after extubation.

Variable	Group	Time after Extubation			
		0 h	1 h	12 h	24 h
Serum cortisol	OR	182 ± 133.67	234 ± 81.17	113.9 ± 51.99	110 ± 83.66
	LS	92.5 ± 36.02	119 ± 62.47 *	88.7 ± 36.18	95.1 ± 93.30
Salivary Cortisol	OR	8 ± 7.6	15 ± 4.1	5.6 ± 1.9	4.5 ± 1.77
	LS	4.9 ± 2.8	11.1 ± 3.8 *	5.2 ± 2.7	4.75 ± 2.71

* Serum cortisol concentration was significantly lower at 1 h in group LS. (*p* < 0.05) * Salivary cortisol concentration was significantly lower at 1 h in the LS group.

**Table 5:** Mean ± SD values of C-Reactive Protein recorded at 0 h, 24 h and 168 h after extubation.

Variable	Group	Time after Extubation		
		0 h	24 h	168 h
C-reactive Protein	OR	17.8 ± 15.8	22.9 ± 11.2	20.8 ± 16.9
	LS	7.1 ± 4.1	18.7 ± 8.2	5.8 ± 3.3 *

* C-Reactive protein concentration was significantly lower at 168 h in group LS. (*p* < 0.05).

## Data Availability

Not applicable.
